# Fast Determination of Satellite-to-Moon Visibility Using an Adaptive Interpolation Method Based on Vertex Protection

**DOI:** 10.3390/s22124451

**Published:** 2022-06-12

**Authors:** Xiaowan Li, Fang Cheng, Pengli Shen, Dongliang Liu

**Affiliations:** 1 National Time Service Center, Chinese Academy of Sciences, Xi’an 710600, China; chengfang@ntsc.ac.cn (F.C.); shenpengli@ntsc.ac.cn (P.S.); liudongliang20@mails.ucas.ac.cn (D.L.); 2 University of Chinese Academy of Sciences, Beijing 100049, China; 3 Key Laboratory of Precise Positioning and Timing Technology, Chinese Academy of Sciences, Xi’an 710600, China

**Keywords:** satellite-to-Moon visibility, rapid determination, adaptive interpolation, vertex protection, lunar navigation

## Abstract

Fast determination of satellite visibility with respect to a target area is important for satellite navigation and positioning. In this paper, we propose an adaptive interpolation algorithm based on vertex protection to solve the satellite visibility period problem more accurately and quickly, where “vertex” refers to the local extremum point. The algorithm can avoid the error in the visibility period calculation caused by skimming the vertices when fitting the multi-hump visibility function under certain fitting accuracy requirements with the traditional adaptive interpolation method. The algorithm does not need to construct a cubic polynomial in each subinterval to determine whether the satellite is visible or not; it only constructs a cubic polynomial to solve the problem if the visibility function of that subinterval is judged to have a solution from the existence theorem of zero points, which can improve the computational efficiency. For the lunar navigation problem, a solution to satellite–Moon visibility calculations based on a vertex-protected adaptive interpolation is given, and the experimental results show that the computation time of the algorithm can be reduced by approximately 98% compared with the brute force method and by approximately 30% compared with the traditional adaptive interpolation algorithm.

## 1. Introduction

Artificial satellites provide important support for global observation services, such as global navigation and remote sensing. As data must be transmitted within a certain visibility range [1,2], the critical foundation for providing services is that the region of interest must be visible from the viewpoint of the satellite. Satellite orbit analysis and constellation coverage analysis are directly related to satellite-to-site visibility [3,4]. The accuracy and speed of satellite coverage are determined by calculating the visible period of the satellite. Task scheduling optimizations, which can reduce service costs by avoiding excessive rescans, require satellite-to-ground visibility predictions [5,6]. At least four satellites need to be used for positioning using navigation satellites, so satellite-to-site visibility is an important indicator of the ability to use satellites for navigation and positioning. Satellite visibility is very important in all aspects of satellite analysis.

The most primitive way to calculate the satellite-to-site visibility is to track the satellite’s trajectory and then to determine the visibility at each moment [5], which is the traditional brute force method. This method is often used as a comparison standard for improving subsequent algorithms. Although the brute force method is accurate, it is very time-consuming. Lawton et al. [7] developed an algorithm for calculating the visible period of a low eccentricity satellite orbit using iteration and a Fourier transform, which greatly improved the calculation speed compared with the brute force method. Alfano et al. [8] used the parabolic blending technique to construct the waveform of the visibility function at equal intervals and used the root of the local cubic polynomial to represent the rise and set times of the satellite. The algorithm is applicable to all satellite orbit types. Sun et al. [9] improved Alfano’s method and proposed an adaptive Hermite interpolation method in order to solve the satellite visibility problem. This method determines the interpolation step by checking the consistency of the second derivative and comparing the extreme value variance. Based on this work, Han et al. [5] derived an adaptive Hermite interpolation technology in a strict sense, which uses the fourth derivative to control the approximation error and achieve a better balance between the accuracy and efficiency. Han et al. [10] used a radial basis function to fit the satellite visibility function and accelerated the calculation of satellite visibility according to the interval contraction strategy. Wan et al. [11] designed a metamodeling framework based on adaptive interpolation and used different metamodel technologies, such as the radial basis function, Kriging, and support vector regression, to solve the satellite visibility problem.

The return to the Moon program has been proposed [12], and it has been realized that precise Moon position information is of great importance for an in-depth exploration of the Moon. In addition, the Magnetospheric Multiscale (MMS) mission proposed by NASA has also confirmed that satellite signals can acquire the position information of spacecraft located beyond the Earth’s orbit [13]. In recent years, the application of Global Navigation Satellite System (GNSS) technology to lunar navigation has attracted the attention of scientists. Kar-Ming et al. [14] simulated lunar navigation with GPS, GLONASS, Galileo, etc., using weak satellite signals for positioning with an accuracy of 200–300 m. The European Space Agency’s proposed Moonlight initiative will carry an advanced satellite signal receiver and perform the first satellite navigation and positioning mission in lunar orbit [15]. In order to carry out navigation and positioning on the Moon, the visibility of the Moon from satellites is an issue that must be analyzed and studied. Both the ESA and NASA have performed detailed analyses of the expected visibility of GNSS signals at Moon altitude [16]. A high-precision and efficient method for determining satellite-to-Moon visibility can provide a theoretical basis for achieving navigation and positioning on the Moon.

Since there are almost no existing algorithms for satellite-to-Moon visibility, this paper draws on the idea of satellite-to-site visibility calculations and identifies a problem when applying the adaptive interpolation algorithm to the satellite-to-Moon visibility calculation (explained in detail in the next section). To avoid this problem, an adaptive interpolation algorithm based on vertex protection is proposed, which is applicable not only to satellite-to-site visibility, but also to satellite-to-Moon visibility. At present, this paper might be the first to provide a fast solution for determining satellite-to-Moon visibility data. In Section 2, the satellite-to-Moon visibility model, which includes the elevation angle model and the Earth occultation model, highlights the drawbacks that arise from directly using the traditional adaptive interpolation algorithm to solve the satellite-to-Moon visibility problem. In Section 3, an adaptive interpolation algorithm based on vertex protection is introduced, and a specific scheme for calculating the satellite-to-Moon visibility using an adaptive interpolation algorithm based on vertex protection is described. In Section 4, the algorithm given in Section 3 is used to experimentally analyze the BDS satellite data. Section 5 concludes this paper.

## 2. Mathematical Models

### 2.1. Elevation Angle Function

Since the radius of the Moon is small, approximately 1738 km on average, and the distance from the satellite to the Moon can reach more than 350,000 km, the Moon is abstracted as a point for analysis in the following model.

Due to the ionospheric effect, tropospheric effect, etc., the satellite must first be considered under the condition of meeting a certain elevation angle with the station in order to achieve a good positioning effect. The elevation angle criterion is thus given as follows:

(1) $$V(t)=\frac{\Delta r\cdot r_{s}}{\Delta r\cdot r_{s}}>\sin\theta_{0}$$

where $\Delta r=r_{m}-r_{s}$ and $r_{s}$ represent the coordinate vectors of the satellite and $r_{m}$ represents the coordinate vector of the Moon. The calculation of the satellite visibility at a certain elevation angle can also be approximated as the problem of solving V(t)−χ=0, where χ is the cosine of elevation angle threshold $\theta_{0}$. The solution to this equation can be used for the time points of the satellite’s rise and set with respect to the Moon.

### 2.2. Earth Occultation

**Figure 1:**
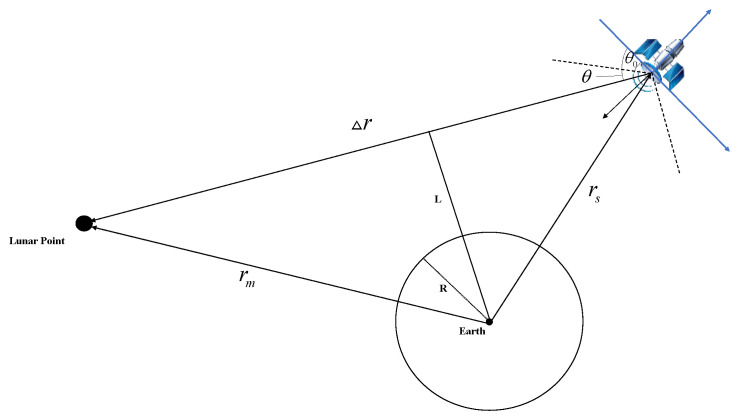
Geometric illustration of satellite-to-lunar visibility.

Since satellites are designed to serve the Earth, they rotate around the Earth and are oriented towards the Earth, so when applying satellites to the Moon, the Earth occultation problem needs to be considered. As shown in Figure 1, when the vertical distance between the satellite and the Moon is less than the radius R of the Earth, the satellite’s line of sight to the Moon is blocked by the Earth, and the satellite is not visible to the Moon. The above problem can be described mathematically as follows:

(2) $$L(t)=\frac{\left||r_{s}\times \Delta r|\right|}{||\Delta r||R}<1$$

where $r_{s}$ is the coordinate vector of the satellite in an Earth-centered, Earth-fixed coordinate system, and $\Delta r=r_{m}-r_{s}$. When L<R, the line of sight from the satellite to the Moon is unobstructed at this time, and it is obscured by the Earth in all other cases.

### 2.3. Adaptive Interpolation Algorithm

**Figure 2:**
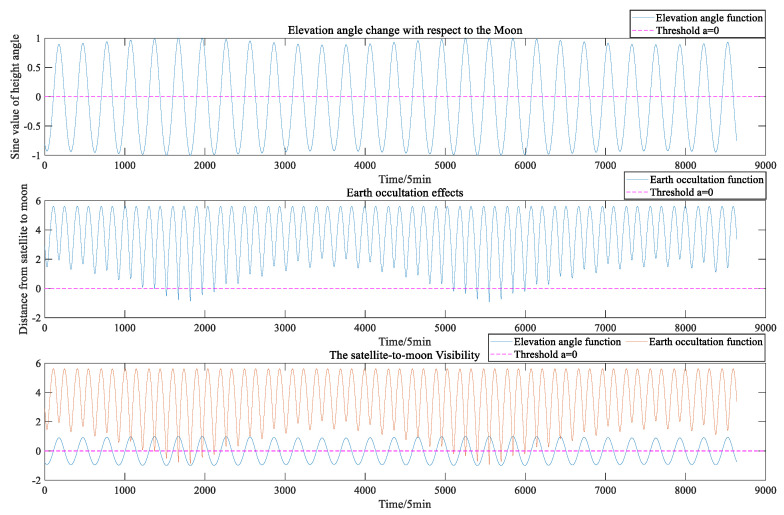
Visibility image of the C01 GEO satellite.

As seen in Figure 2, where C01 represents a BDS GEO satellite, both the rise and set functions of the satellite-to-Moon visibility and the period function of the Earth occultation between the Earth and the Moon are multi-hump functions, which are irregular and do not have specific analytical expressions. It is known from previous work [5,10] that the solution to a similar function can be approximated using the idea of segment interpolation.

For the satellite-to-Moon visibility, this paper utilizes the idea of segmented Hermite interpolation to construct a cubic polynomial between the subintervals $t_{0}$ to $t_{1}$ and calculates the maximum step from the second-order derivatives of the corresponding visibility functions, as well as the visibility function values in satisfying a certain error limit. According to [17], the function of segmented cubic Hermite interpolation can be written as:

(3) $$\begin{array}{l} S(t)=f(t_{0})+f^{\prime }(t_{0})(t-t_{0})+a{\left(t-t_{0}\right)}^{2}+b{\left(t-t_{0}\right)}^{3}\\ a=(3\frac{f(t_{1})-f(t_{0})}{h^{2}}-\frac{f^{\prime }(t_{1})+2f^{\prime }(t_{0})}{h})\\ b=(\frac{f^{\prime }(t_{1})+f^{\prime }(t_{0})}{h^{2}}-2\frac{f(t_{1})-f(t_{0})}{h^{3}}) \end{array}$$

where $t_{0}$ denotes the starting point for constructing the cubic polynomial, $t_{1}$ denotes the end point, and $h=t_{1}-t_{0}$. When the fourth-order derivative exists, the approximation error can be described by:

(4) $$R(t)=V(t)-S(t)=\frac{V^{\left(4\right)}(\eta )}{4!}{\left((t-t_{0})(t-t_{1})\right)}^{2}$$

where $t,\eta \in \left[t_{0},t_{1}\right]$, R(t), denotes the interpolation error residual term, V(t) denotes the true function, $V^{\left(4\right)}(\eta )$ is the fourth-order derivative of V(t) and can be obtained using higher-order Hermite interpolation, and S(t) denotes the constructed cubic polynomial. The quadratic polynomial of the function can be approximated by the following equation:

(5) $$\begin{array}{l} V^{\left(4\right)}(t)\approx 120\kappa_{5}t+24\kappa_{4}\\ \kappa_{4}=\frac{4}{h^{4}}\alpha -\frac{4}{h^{4}}\beta -\frac{24}{h^{5}}\chi \\ \kappa_{5}=\frac{24}{h^{5}}\left[V(t_{0})-V(t_{1})\right]+\frac{4}{h^{4}}\left[\dot{V}(t_{0})-\dot{V}(t_{0}+\frac{h}{2})+\dot{V}(t_{1})\right]\\ \alpha =\left[V(t_{0})+4V(t_{0}+\frac{h}{2})+V(t_{1})\right]\\ \beta =\left[\dot{V}(t_{0})(2t_{0}+3t_{1})+10\dot{V}(t_{0}+\frac{h}{2})(t_{0}+t_{1})+3\dot{V}(t_{1})(3t_{0}+2t_{1})\right]\\ \chi =\left[V(t_{0})(2t_{0}+3t_{1})-V(t_{1})(3t_{0}+2t_{1})\right] \end{array}$$

Using $V^{\left(4\right)}(t)$ to place limits on the errors, the following is obtained:

(6) $$|R(t){|}_{\max}\leq |\frac{V^{\left(4\right)}{\left(n\right)}_{\max}}{4!}|{\left[(t-t_{0}){\left(t-t_{1}\right)}^{2}\right]}_{\max}\leq |5a_{5}n+a_{4}{|}_{\max}{\left(\frac{h^{2}}{4}\right)}^{2}$$

Then, the step is:

(7) $$\hat{h}={\left(\frac{16\varepsilon }{|5\kappa_{5}\eta +\kappa_{4}{|}_{\max}}\right)}^{1/4}$$

The solution for the step is an iterative process, and the termination condition of the iterative solution is:

(8) $$\frac{\left|{\hat{h}}_{k}-{\hat{h}}_{k-1}\right|}{{\hat{h}}_{k-1}}\leq u$$

From the above equation, it is clear that the first-order derivative of the original function is required to perform the segmented Hermite interpolation, where the first-order derivative of the satellite elevation angle is:

(9) $$\dot{V}(t)=\frac{1}{\left\|\Delta r\right\|}(\Delta \dot{r}\cdot r_{s}+\Delta r\cdot {\dot{r}}_{s})-\frac{1}{\left\|\Delta r\right\|}(\Delta r\cdot \Delta \dot{r})\Delta r\cdot r_{s}$$

The first-order derivative of the Earth occultation model is:

(10) $$\dot{L}(t)=\frac{\left(r_{s}\times \Delta r)\cdot ({\dot{r}}_{s}\times \Delta r+r_{s}\times \Delta \dot{r}\right)}{\left\|r_{s}\times \Delta r\|\cdot \|\Delta \right\|}-\frac{\Delta \cdot \dot{\Delta }\cdot \|r_{s}\times \Delta r\|}{\|\Delta {\|}^{3}}$$

where $\Delta \dot{r}=v_{m}-v_{s}$.

## 3. Algorithm Design

### 3.1. Vertex Protection Algorithm

**Figure 3:**
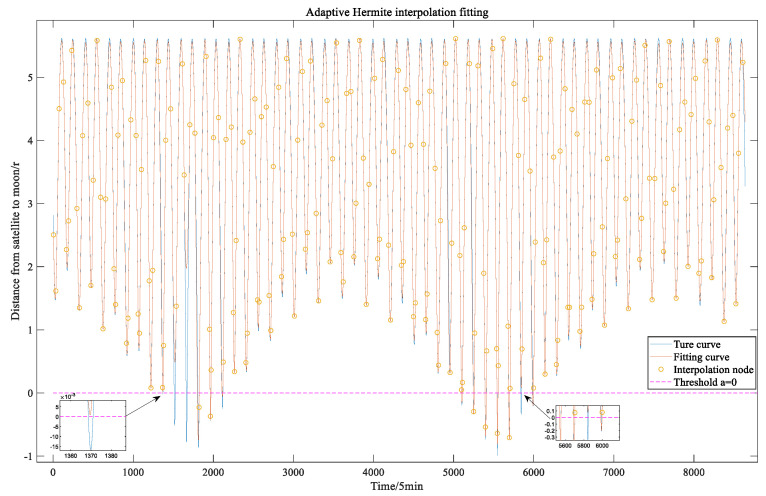
Conventional adaptive algorithm to fit the occlusion function.

The satellite-to-Moon visibility consists of two aspects: the availability of the satellite signal is limited by the elevation angle (as deduced in Section 2.1), and the Earth may obscure the line of sight from the satellite to the Moon (as deduced in Section 2.2). The algorithm provided in Section 2.3 can calculate the maximum interpolation step while guaranteeing the fitting accuracy. As shown in Figure 3, under the low fitting accuracy requirement, when calculating whether the Earth obscures the line of sight between the satellite and the Moon, an intersection exists between the original visibility function curve and the threshold, but the fitted curve has no intersection with the threshold, which causes errors when calculating the Earth occultation period in one cycle (28 days). Such a problem is related to the threshold selection and fitting accuracy requirement.

To avoid this problem, in this paper, the basic idea is to try to make the local extremal points of the multi-hump function the endpoints of the subinterval, where the local extremal points are the “vertices”. This method not only avoids the visibility calculation error, but also has the advantage that it can directly determine whether there is a solution to the original function within that subinterval without constructing a cubic polynomial in each subinterval. The interpolation method must know the original function values of the two endpoints. Then, according to the existence theorem of zero points, only when the two endpoints’ values corresponding to the function are different must the subinterval exist within the zero solution. Then, a cubic polynomial is constructed and solved. The vertex protection algorithm is designed as follows:

(1) Calculate the adaptive step $h_{0}$ according to the algorithm in Section 2.3, determine the termination time $t_{e}$ according to step $h_{0}$ and start time $t_{s}$, and introduce the search factor σ.

(2) Calculate the values $f_{1}$, $f_{2}$, $f_{3}$, and $f_{4}$ of the function F(t) at $t_{1}=t_{s}$, $t_{2}=t_{s}+\sigma$, $t_{3}=t_{e}-\sigma$, and $t_{4}=t_{e}$ based on the original function (satellite rise and set function or occultation function, respectively).

(3) Compare the magnitudes of $f_{1}$, $f_{2}$, and $f_{4}$ or $f_{1}$, $f_{3}$, and $f_{4}$. When $f_{2}$ is smaller than both $f_{1}$ and $f_{4}$, go to step (4), and when $f_{3}$ is larger than both $f_{1}$ and $f_{4}$, go to step (5). When $f_{2}$ is greater than both $f_{1}$ and $f_{4}$, go to step (6), and when $f_{3}$ is smaller than both $f_{4}$ and $f_{4}$, go to step (7).

(4) Let $t_{4}=t_{2}$, and calculate the corresponding $f_{4}$. Calculate the corresponding $f_{2}$ when $t_{2}=t_{2}+\sigma$. Compare $f_{4}$ and $f_{2}$. If $f_{2}<f_{4}$, repeat step (4); otherwise, go to step (8).

(5) Let $t_{4}=t_{3}$, and calculate the corresponding $f_{4}$. Calculate the corresponding $f_{3}$ when $t_{3}=t_{3}-\sigma$. Compare $f_{4}$ and $f_{3}$. If $f_{3}>f_{4}$, repeat step (5); otherwise, go to step (8).

(6) Let $t_{4}=t_{2}$, and calculate the corresponding $f_{4}$. Calculate the corresponding $f_{2}$ when $t_{2}=t_{2}+\sigma$. Compare $f_{2}$ and $f_{4}$. If $f_{2}>f_{4}$, repeat step (4); otherwise, go to step (8).

(7) Let $t_{4}=t_{3}$, and calculate the corresponding $f_{4}$. Calculate the corresponding $f_{3}$ when $t_{3}=t_{3}-\sigma$. Compare $f_{4}$ and $f_{3}$. If $f_{3}<f_{4}$, repeat step (5); otherwise, go to step (8).

(8) Calculate when $t_{s}=t_{1}$ and $t_{e}=t_{4}$ correspond to $f_{s}=f_{1}$ and $f_{e}=f_{4}$ and obtain $h=t_{e}-t_{s}$.

Here, σ denotes the step for searching for the local maxima of the multi-hump function, which determines the search accuracy and efficiency. The smaller σ is, the more accurate and slower the local maxima of the multi-hump function will be as the endpoint of the interpolated subinterval; the larger σ is, the coarser and faster the local maxima of the multi-hump function will be as the endpoint of the interpolated subinterval.

### 3.2. Adaptive Interpolation Based on Vertex Protection

**Figure 4:**
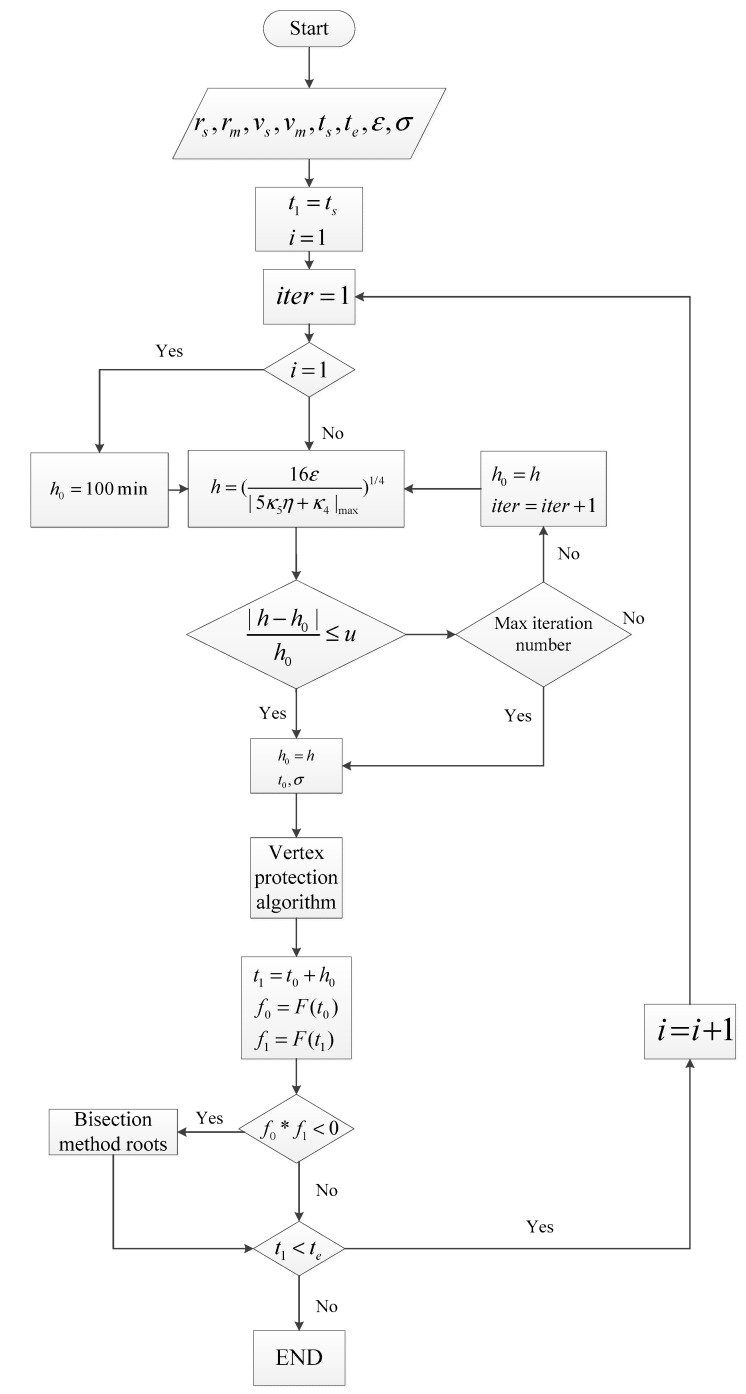
Flowchart of the adaptive interpolation based on the vertex protection algorithm.

The basic idea of adaptive interpolation based on the vertex protection method is as follows: according to the interpolation error function and the fourth-order derivative constraints, calculate the interpolation step $h_{0}$, and then use the obtained step $h_{0}$ and the starting time $t_{s}$ in the vertex protection algorithm to obtain h. Next, try to make the vertex of the multi-hump function the interpolation endpoint, and then, according to step h and the starting time point $t_{0}$, determine the termination time point $t_{1}$ of the subinterval. The values $f_{0}$ and $f_{1}$ corresponding to the original function F(t) at times $t_{0}$ and $t_{1}$, respectively, are calculated to determine whether the original function in this region has a solution. This shortens the process of constructing a cubic polynomial function for each subinterval in order to determine whether there is a solution and then solving for it, thereby effectively reducing the amount of computation. For the satellite-to-Moon visibility problem, the step calculation is based on the mathematical model in Section 2.3, and the vertex protection algorithm is the method proposed in Section 3.1. The rise and set periods of the satellite relative to the Moon are quickly solved using the adaptive interpolation method based on vertex protection, and then the Earth’s occultation for the line of sight between the satellite and the Moon is calculated within such periods, which can shorten the Earth occultation calculated time. The specific computational flow chart of the adaptive interpolation based on vertex protection is shown in Figure 4.

## 4. Experiment and Analysis

Since the period of the Moon’s revolution around the Earth is approximately 27.32 days, the period of a geosynchronous Earth orbit (GEO) satellite is approximately 24 h, the period of an inclined geosynchronous orbit (IGSO) satellite is approximately 24 h, and the period of a medium Earth orbit (MEO) satellite is approximately 12 h. Satellite data with a period of 28 days for the Moon’s revolution are used for the analysis. The data used here are from the multisystem precision ephemeris file provided by the Analysis Centre of Wuhan University for 31 December–30 January 2021, which contains the coordinates of each BDS, GLONASS, and Galileo satellite at five-minute intervals in the geocentric geostationary coordinate system. The lunar coordinates were calculated using a simplified model [18].

**Figure 5:**
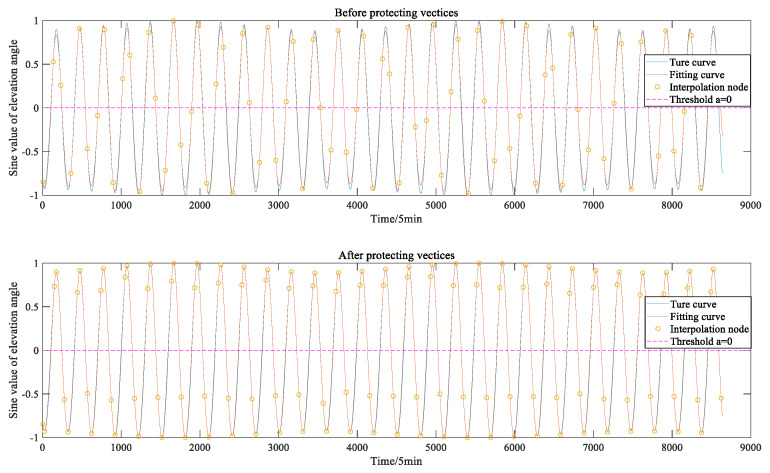
Effects of vertex protection algorithms on fitting of elevation angle functions.

**Figure 6:**
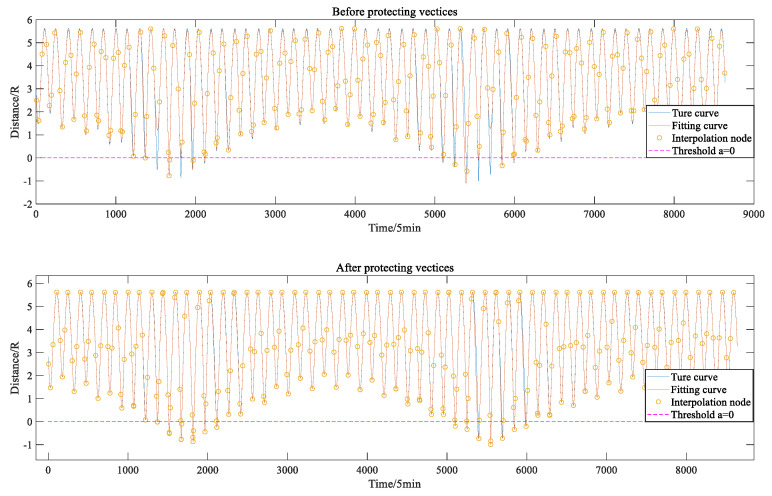
Effects of vertex protection algorithms on fitting of Earth occultation functions.

Figure 5 and Figure 6 show a comparison between the adaptive interpolation algorithm based on vertex protection and the traditional adaptive interpolation algorithm [5] in fitting the elevation angle function and the Earth occlusion function from the C01 satellite to the Moon when ε=0.1 and σ=0.2. From the two figures, it can be seen that the adaptive interpolation algorithm based on vertex protection can make the local extreme points of the multi-hump functions, such as the elevation angle function and the Earth occlusion function, the endpoints of the interpolation sub-interval (to the greatest extent possible), and the fit is good.

The period of visibility from the satellite to the Moon is calculated as the true value using the brute force method, including the solution for the rise and set moments of the satellite with respect to the Moon and the period of the Earth’s line-of-sight obscuration between the satellite and the Moon. 

Table 1 shows a comparison between the adaptive interpolation based on the vertex protection algorithm, the conventional adaptive interpolation algorithm, and the brute force method in solving the number of periods of visibility between the satellite and the Moon; that is, the times of the satellite’s rise and set with respect to the Moon and the times of the Earth’s occultation. For example, for the C01 satellite at fitting errors ε=0.1 and ε=0.01, the C06 satellite at fitting error ε=0.1, and the C11 satellite at fitting error ε=0.1, the solved Earth occlusion times do not match the real times, which are errors that cannot occur in practical applications. Adaptive interpolation based on vertex protection can accurately solve the Earth occlusion count for satellites C01, C06, and C11 when the search factor in the protection vertex algorithm is σ=0.2 for the fitting errors ε=0.1, ε=0.01, and ε=0.001.

The efficiency of the adaptive interpolation method based on vertex protection proposed in this paper is compared to that of the traditional adaptive interpolation method when applied to the satellite-to-Moon visibility calculation in Table 2, Table 3 and Table 4. For satellites C01, C06, and C11, under the limits of interpolation accuracies of 0.1, 0.01, and 0.001, respectively, the number of step calculations, the number of times the algorithm actually constructs a cubic polynomial for the solution, and the time efficiency improvement (compared with the brute force method) are compared.

As seen from these tables, the number of step calculations is always lower than that of the traditional adaptive interpolation algorithm, and the difference in the number of step calculations is more obvious as the fitting accuracy decreases. The algorithm proposed in this paper constructs a cubic polynomial for the number of solutions, and a cubic polynomial is constructed for when there is a solution in that subinterval; however, the traditional adaptive interpolation algorithm requires the construction of cubic polynomials in each subinterval to determine whether the interval has a solution and then calculate it. These tables give the percentage improvements in the time efficiency for adaptive interpolation based on the vertex protection algorithm and traditional adaptive interpolation compared with the brute force method. Under the requirement of a low fitting accuracy ε=0.1, the computational efficiency of the algorithm proposed in this paper and the traditional algorithm are essentially comparable, and both improve by approximately 98% compared with the brute force method. The higher the requirement is, the more obvious the improvement in efficiency. When ε=0.001 and σ=0.2, it can be seen from the following table that, for the C11 MEO satellite, the algorithm proposed in this paper improves the computational efficiency by approximately 30% compared with the traditional adaptive computation. This affects the accuracy of the vertex protection algorithm when seeking the vertex as the endpoint of the fitted subinterval, where the larger σ is, the faster the algorithm is computed under the condition that the overall cycle is computed error-free.

To represent the accuracy of the adaptive interpolation algorithm based on vertex protection, the percentage normalization error defined by [19] is listed, where the percentage normalization is specifically defined as follows:

(11) $$PNE=\frac{\left|Predicted\;satellite\;rise/set\;time-Acutal\;satellite\;rise/set\;time\right|}{Actual\;in-view\;period}\times 100$$

**Figure 7:**
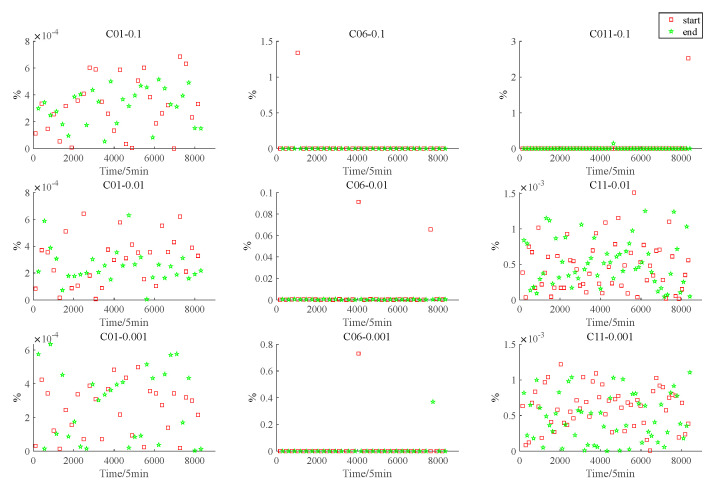
Graphs of C01, C06, and C11 satellite rise and set times.

**Figure 8:**
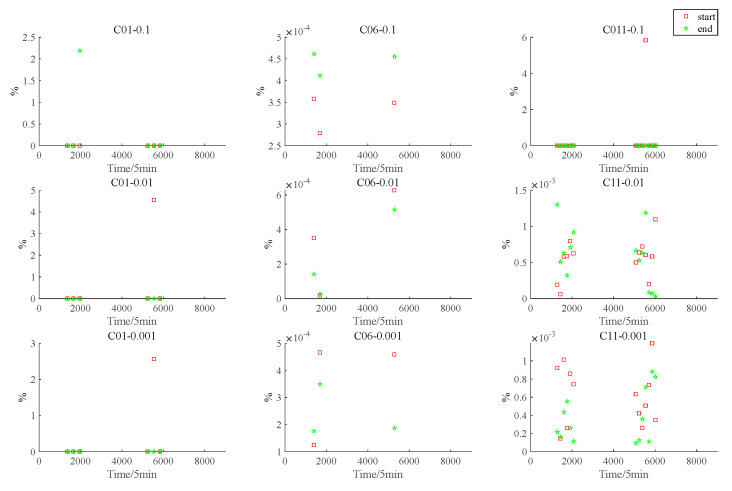
Graphs of Earth occultation start and end times.

Figure 7 and Figure 8 show the PNE of the satellite rise and set times and the PNE of the Earth occultation start and end times, respectively; the red squares indicate the PNE of the start time and the green stars indicate the PNE of the end time. The subgraph C01-0.1 in Figure 7 shows the PNE of the C01 satellite at the rise and set times relative to the Moon when ε=0.1. Other subgraphs can also be interpreted in this way. The subgraph C01-0.1 in Figure 8 indicates the PNE values at the start and end times of the Earth occultation for the line of sight from satellite C01 to the Moon when ε=0.1. Other subgraphs are also expressed in this way.

## 5. Conclusions

Due to the existence of the traditional adaptive interpolation de-fitting error, the solution to the satellite visibility problem results in a cycle calculation error. In this paper, we introduced an adaptive interpolation algorithm based on vertex protection that makes the vertices of the multi-hump function the endpoints of the adaptive interpolation subinterval to the greatest extent possible in order to avoid the period calculation error in the satellite visibility problem, making the adaptive interpolation algorithm applicable to a wider range of satellite visibility problems. The first solution for quickly determining the satellite-to-Moon visibility problem was given, and experiments were conducted using data from three BDS orbiting satellites to analyze its efficiency and accuracy. The experiments showed that the method reduces the computation time by approximately 98% compared to the brute force method, and the computational accuracy and efficiency of the algorithm proposed in this paper are better than those of the traditional adaptive interpolation method, with a reduction in computation time of up to 30%.

## Figures and Tables

**Table 1:** Satellite visibility times.

Satellite	Methods	ε	Rise and Set Times	Occlusion Times
C01 GEO Satellite	Brute force method	0.1	28	6
		0.01	28	6
		0.001	28	6
	Self-adaptive interpolation technique without vertex protection	0.1	28	4
		0.01	28	5
		0.001	28	6
	Self-adaptive interpolation technique with vertex protection	0.1	28	6
		0.01	28	6
		0.001	28	6
C06 IGSO Satellite	Brute force method	0.1	28	3
		0.01	28	3
		0.001	28	3
	Self-adaptive interpolation technique without vertex protection	0.1	27	1
		0.01	28	3
		0.001	28	3
	Self-adaptive interpolation technique with vertex protection	0.1	28	3
		0.01	28	3
		0.001	28	3
C11 MEO Satellite	Brute force method	0.1	53	13
		0.01	53	13
		0.001	53	13
	Self-adaptive interpolation technique without vertex protection	0.1	53	12
		0.01	53	13
		0.001	53	13
	Self-adaptive interpolation technique with vertex protection	0.1	53	13
		0.01	53	13
		0.001	53	13

**Table 2:** Comparison of the C01 satellite visibility solving efficiencies.

Case	ε=0.1 After/Before		ε=0.01 After/Before		ε=0.001 After/Before	
Step calculation times	248	295	404	539	1104	1611
Resolution times	71	295	69	539	70	1611
Run time	98.34%	98.8%	98.03%	98.2%	96.5%	94.6%

**Table 3:** Comparison of the C06 satellite visibility solving efficiencies.

Case	ε=0.1 After/Before		ε=0.01 After/Before		ε=0.001 After/Before	
Step calculation times	218	239	472	745	2990	6083
Resolution times	79	239	63	745	63	6083
Run time	98.6%	99.2%	98.1%	97.6%	93.5%	84.6%

**Table 4:** Comparison of the C11 satellite visibility solving efficiencies.

Case	ε=0.1 After/Before		ε=0.01 After/Before		ε=0.001 After/Before	
Step calculation times	467	579	1318	2196	10,539	19,282
Resolution times	135	579	135	2196	135	19,282
Run time	97.5%	98.2%	95.0%	93.6%	76.2%	47.6%

## Data Availability

All data are available from the Multi-GNSS Experiment (MGEX).

## References

[1] Jin S. (2013). Recent progresses on Beidou/COMPASS and other global navigation satellite systems (GNSS)—I. Adv. Space Res..

[2] Yang D., Yang J., Xu P. (2016). Timeslot scheduling of inter-satellite links based on a system of a narrow beam with time division. GPS Solut..

[3] Li Y.J., Zhao S.H., Wu J.L. (2016). A general evaluation criterion for the coverage performance of LEO constellations. Aerosp. Sci. Technol..

[4] Ulybyshev Y. (2015). Geometric Analysis of Low-Earth-Orbit Satellite Communication Systems: Covering Functions. J. Spacecr. Rocket..

[5] Han C., Gao X.J., Sun X.C. (2017). Rapid Satellite-to-Site Visibility Determination Based on Self-Adaptive Interpolation Technique. Sci. China Technol. Sci..

[6] Wang J., Zhu X., Qiu D., Yang L.T. (2014). Dynamic Scheduling for Emergency Tasks on Distributed Imaging Satellites with Task Merging. IEEE Trans. Parallel Distrib. Syst..

[7] Lawton A.J. (2015). Numerical method for rapidly determining satellite-satellite and satellite-ground station in-view periods. J. Guid. Control. Dyn..

[8] Alfano S., Negron D., Moore J.L. (1992). Rapid Determination of Satellite Visibility Periods. J. Astronaut. Sci..

[9] Sun X., Cui H., Han C., Tang G. (2012). Apchi Technique for Rapidly and Accurately Predicting Multi-Restriction Satellite Visibility. https://www.researchgate.net/profile/Xiucong-Sun-2/publication/270892862_APCHI_Technique_for_Rapidly_and_Accurately_Predicting_Multi-Restriction_Satellite_Visibility/links/5527bada0cf2779ab78a9b1c/APCHI-Technique-for-Rapidly-and-Accurately-Predicting-Multi-Restriction-Satellite-Visibility.pdf.

[10] Chao H., Yang P., Wang X., Liu S. A Fast Computation Method for the Satellite-to-Site Visibility. Proceedings of the 2018 IEEE Congress on Evolutionary Computation (CEC).

[11] Wang X., Han C., Yang P., Sun X. (2019). Onboard satellite visibility prediction using metamodeling based framework. Aerosp. Sci. Technol..

[12] NASA Artemis Program. https://www.nasa.gov/specials/artemis/.

[13] https://www.nasa.gov/feature/goddard/2019/record-breaking-satellite-advances-nasa-s-exploration-of-high-altitude-gps.

[14] Cheung K.M., Lee C., Heckman D. Feasibility of “Weak GPS” Real-Time Positioning and Timing at Lunar Distance. Proceedings of the 2020 IEEE Aerospace Conference.

[15] Schönfeldt M., Grenier A., Delépaut A., Swinden R., Giordano P., Ventura-Traveset J. (2020). Across the Lunar Landscape, Space Exploration with GNSS Technology. InsideGNSS.

[16] Delépaut A., Giordano P., Ventura-Traveset J., Blonski D., Schönfeldt M., Schoonejans P., Aziz S., Walker R. (2020). Use of GNSS for lunar missions and plans for Lunar In-Orbit Development. Adv. Space Res..

[17] Süli E., Mayers D.F. (2003). An Introduction to Numerical Analysis.

[18] Montenbruck O., Gill E. (2000). Satellite Orbits.

[19] Ali I., Al-Dhahir N. (1999). Predicting the visibility of LEO satellites. IEEE Trans. Aerosp. Electron. Syst..

